# Analysis of *SLX4/FANCP *in non-*BRCA1/2*-mutated breast cancer families

**DOI:** 10.1186/1471-2407-12-84

**Published:** 2012-03-08

**Authors:** Juana Fernández-Rodríguez, Francisco Quiles, Ignacio Blanco, Alex Teulé, Lídia Feliubadaló, Jesús del Valle, Mónica Salinas, Àngel Izquierdo, Esther Darder, Detlev Schindler, Gabriel Capellá, Joan Brunet, Conxi Lázaro, Miguel Angel Pujana

**Affiliations:** 1 Hereditary Cancer Program, Catalan Institute of Oncology (ICO), Hospital Duran i Reynals, Bellvitge Institute for Biomedical Research (IDIBELL), L'Hospitalet, Barcelona, Catalonia, Spain; 2 Hereditary Cancer Program, ICO, Hospital Josep Trueta, Girona Biomedical Research Institute (IdIBGi), Girona, Catalonia, Spain; 3 Department of Human Genetics, University of Würzburg, Biozentrum, Würzburg, Germany; 4 Translational Research Laboratory, Biomedical Research Center Network for Epidemiology and Public Health (CIBERESP), ICO, IDIBELL, L'Hospitalet, Barcelona, Catalonia, Spain

## Abstract

**Background:**

Genes that, when mutated, cause Fanconi anemia or greatly increase breast cancer risk encode for proteins that converge on a homology-directed DNA damage repair process. Mutations in the *SLX4 *gene, which encodes for a scaffold protein involved in the repair of interstrand cross-links, have recently been identified in unclassified Fanconi anemia patients. A mutation analysis of *SLX4 *in German or Byelorussian familial cases of breast cancer without detected mutations in *BRCA1 *or *BRCA2 *has been completed, with globally negative results.

**Methods:**

The genomic region of *SLX4*, comprising all exons and exon-intron boundaries, was sequenced in 94 Spanish familial breast cancer cases that match a criterion indicating the potential presence of a highly-penetrant germline mutation, following exclusion of *BRCA1 *or *BRCA2 *mutations.

**Results:**

This mutational analysis revealed extensive genetic variation of *SLX4*, with 21 novel single nucleotide variants; however, none could be linked to a clear alteration of the protein function. Nonetheless, genotyping 10 variants (nine novel, all missense amino acid changes) in a set of controls (138 women and 146 men) did not detect seven of them.

**Conclusions:**

Overall, while the results of this study do not identify clearly pathogenic mutations of *SLX4 *contributing to breast cancer risk, further genetic analysis, combined with functional assays of the identified rare variants, may be warranted to conclusively assess the potential link with the disease.

## Background

A functionally coherent network of gene and/or protein interactions, altered in Fanconi anemia (FA) and breast cancer (BrCa), has emerged in recent years [1]. Fifteen genes that, when mutated, cause FA (*FANC *genes) and several genes that may harbor mutations of high, moderate or low penetrance for BrCa risk encode for proteins that converge on a homology-directed DNA damage repair process [2]. As further evidence of a fundamental common causal basis between these diseases, germline bi- and mono-allelic loss-of-function mutations in four of these genes cause FA and BrCa, respectively: *FANCD1/BRCA2 *[3,4], *FANCJ/BRIP1 *[5-8], *FANCN/PALB2 *[9-11] and *FANCO*/*RAD51C *[12,13] (more recent data suggests that mutations in *RAD51C *may be primarily linked to ovarian cancer risk [14]). This evidence marks any novel gene involved in the aforementioned network or process as a candidate to harbor mutations in unclassified FA and/or BrCa patients.

Interstrand DNA cross-link agents, such as mitomycin-C used in diagnostic tests for FA, block replication forks and may therefore cause genome instability. Homologs of SLX4 in model organisms were initially identified as necessary for replication fork restart following exposure to DNA-damaging agents [15]. Subsequently, SLX4 homologs have been shown to play a key role as docking molecules for the repair of interstrand cross-links [16,17]. These observations pointed to the human *SLX4 *gene as a *FANC *candidate for unclassified patients and, as a result, two groups have recently described mutations (renamed *FANCP *gene or FA-P subtype) [18,19]. The potential link to BrCa risk has been examined, to date, in 52 German or Byelorussian patients with familial breast cancer [20]: the study has not revealed truncating or clearly pathogenic mutations, but has identified four unclassified missense variants. Here, we conducted a more detailed study of the *SLX4 *gene in 94 index BrCa cases from Spanish families negative for *BRCA1 *and *BRCA2 *mutations. As recently reported [20], our results do not show truncating or clearly pathogenic mutations, although they do describe seven missense variants of unknown biological significance that are not found in controls.

## Methods

### Study samples

Since its creation in 1999, the Hereditary Cancer Program at the ICO has identified a set of high-risk families with suspected hereditary breast and/or ovarian cancer syndrome. Following the Catalan Consensus Onco Guidelines on genetic testing for this condition, patients are analysed for mutations in the *BRCA1 *or *BRCA2 *genes after receiving appropriate genetic counselling and providing written informed consent. This genetic analysis consists of screening for point mutations and large rearrangements affecting those genes. For the present study, a total of 94 affected individuals belonging to 94 unrelated families negative for *BRCA1 *or *BRCA2 *mutations were selected. In addition to negativity for mutations in *BRCA1 *or *BRCA2*, the inclusion criteria were: at least three first-degree relatives affected by breast or ovarian cancer; or at least two first-degree female relatives affected by breast cancer (at least one of them diagnosed before the age of 50); or at least one case of female breast cancer plus at least one case of either ovarian, female bilateral breast, or male breast cancer. Among the selected cases, 10 families were represented with an elevated prior risk of harboring a high-penetrance mutation, as calculated with the BRCAPRO [21] algorithm (briefly, 94 families mean = 0.40, standard deviation = 0.26, 95% confidence interval 0.11-0.99). Control samples, consisting of 138 women and 146 men, were taken from a hospital-based cancer association study (a detailed description of the study population, composition and interviews has been given elsewhere [22]). Specifically, these individuals were randomly enrolled from non-cancer patients admitted to the same general hospital as the BrCa cases. To avoid selection bias, the inclusion criterion for controls was that the current admission to the hospital should be for a new disease (not previously diagnosed). The studies were approved by the IDIBELL ethics committee and participants gave written informed consent for their participation, and for the genetic analysis of their biological samples, according to the Declaration of Helsinki.

### Mutation analysis

The *SLX4 *exons and exon-intron boundaries were sequenced from polymerase chain reactions using previously defined primers and conditions [18]; for exon 7, AmpliTaq Gold DNA Polymerase (Applied Biosystems) and 10% of dimethyl sulfoxide were used. The reaction products were purified from remaining primers using ExoSAP-IT (GE Healthcare) and sequencing reactions performed following a standard Sanger method with the BigDye Terminator v3.1 Cycle Sequencing Kit (Applied Biosystems). Samples harboring DNA variants were re-sequenced at least once using an independent DNA aliquot from the first-pass analysis. The guidelines of the Human Genome Variation Society and the reference sequences NM_032444.2 and NP_115820.2 of the National Center for Biotechnology Information were used for nomenclature.

### Genotyping

Assays based on the KASPar technology were performed following the manufacturer's instructions (KBioscience). Reactions were carried out in a 384-well format with 2% of duplicates, and negative and positive (i.e. BrCa patient carrier) sample controls present in each plate.

## Results and discussion

The genomic region of *SLX4*, comprising all exons and exon-intron boundaries, was sequenced in 94 BrCa familial cases that match a criterion indicating the potential presence of a highly-penetrant germline mutation, following exclusion of *BRCA1 *or *BRCA2 *mutations (see Methods). This mutational analysis revealed 49 variants: 21 novel and 28 which are currently annotated in the single nucleotide polymorphism database (dbSNP [23]) (Table 1). Of the 49 variants, 21 were found only once, which include three changes identified by the 1,000 Genomes Project [24] (Table 1): rs72778139-T has no known frequency data; rs76488917-A has an allele frequency of 0.02 in Caucasians; and rs115694169-A has an allele frequency of 0.03 in the Yoruba people of Ibadan (there is no data for Caucasians). Excluding these from the set of 21 with low frequency revealed eight missense and five silent changes at the protein level, and five intronic changes (Table 1). A neural network splicing prediction [25] model did not strongly support alteration by any of the identified intronic variants (data not shown). Together, these results suggest extensive genetic variation at the *SLX4 *locus among individuals in our population, but provide no obvious link to BrCa risk.

**Table 1:** *SLX4 *variants found in non-*BRCA1/2*-mutated familial BrCa cases

Exon	Nucleotide change	Change type	Amino acid change	Number of carriers*			dbSNP†
					
				Het (%)	Hom (%)	Total	
2	c.248G > C	Missense	p.Gly83Ala	1 (1.1)	0	94	NA
2	c.339T > C	Silent	p. =	1 (1.1)	0	94	NA
2	c.421G > T	Missense	p.Gly141Trp	1 (1.1)	0	94	NA
3	c.555C > T	Silent	p. =	9 (9.5)	0	94	rs74640850
3	c.610C > T	Missense	p.Arg204Cys	10 (10.6)	0	94	rs79842542
3	c.678C > T	Silent	p. =	3 (3.2)	0	94	rs28516461
3	c.590T > C	Missense	p.Val197Ala	1 (1.1)	0	94	NA
3	c.710G > A	Missense	p.Arg237Gln	2 (2.1)	0	94	NA
3	c.753G > A	Silent	p. =	24 (25.5)	2 (2.1)	94	rs8061528
4	c.761-32T > G	Intronic	p. =	2 (2.1)	0	94	NA
5	c.1152A > G	Silent	p. =	11 (11.7)	0	94	rs112511042
5	c.1153C > A	Missense	p.Pro385Thr	1 (1.1)	0	94	rs115694169
5	c.1156A > G	Missense	p.Met386Val	11 (11.7)	0	94	rs113490934
5	c.1163 + 10C > T	Intronic	p. =	11 (11.7)	0	94	rs80116508
6	c.1164-16T > C	Intronic	p. =	1 (1.1)	0	94	NA
6	c.1164-40C > A	Intronic	p. =	1 (1.1)	0	94	NA
6	c.1164-66T > A	Intronic	p. =	2 (2.1)	0	94	NA
6	c.1164-75C > G	Intronic	p. =	11 (11.7)	0	94	rs59622164
6	c.1366 + 11T > C	Intronic	p. =	12 (12.8)	0	94	rs76350200
7	c.1371T > G	Missense	p.Asn457Lys	10 (10.6)	0	94	rs74319927
7	c.1419C > T	Silent	p. =	1 (1.1)	0	94	NA
8	c.1846G > A	Missense	p.Val616Met	1 (1.1)	0	94	NA
9	c.2012T > C	Missense	p.Leu671Ser	11 (11.8)	0	93	rs77985244
9	c.2013 + 23G > A	Intronic	p. =	11 (11.7)	0	94	rs112226642
9	c.2013 + 137G > C	Intronic	p. =	11 (11.7)	0	94	rs80186343
10	c.2160 + 50C > T	Intronic	p. =	10 (10.6)	0	94	rs75762935
12	c.2346C > T	Silent	p. =	1 (1.1)	0	94	NA
12	c.2469G > C	Missense	p.Trp823Cys	1 (1.1)	0	94	NA
12	c.2854G > A	Missense	p.Ala952Thr	8 (8.5)	0	94	rs59939128
12	c.2855C > T	Missense	p.Ala952Val	8 (8.5)	0	94	rs78637028
12	c.3162G > A	Silent	p. =	1 (1.1)	0	94	rs76488917
12	c.3365C > T	Missense	p.Pro1122Leu	12 (12.8)	1 (1.1)	94	rs714181
12	c.3662C > T	Missense	p.Ala1221Val	10 (10.6)	0	94	rs3827530
12	c.3812C > T	Missense	p.Ser1271Phe	4 (4.2)	0	94	rs3810813
12	c.3872C > T	Missense	p.Thr1291Met	1 (1.1)	0	94	NA
12	c.4261A > T	Missense	p.Ile1421Phe	1 (1.1)	0	94	NA
12	c.4409C > T	Missense	p.Pro1470Leu	1 (1.1)	0	94	rs72778139
12	c.4500T > C	Silent	p. =	42 (44.7)	21 (22.3)	94	rs3810812
12	c.4530G > T	Silent	p. =	1 (1.1)	0	94	NA
13	c.4637-125C > T	Intronic	p. =	1 (1.1)	0	94	NA
13	c.4637-227C > T	Intronic	p. =	9 (9.6)	0	94	rs75693937
13	c.4739 + 10C > T	Intronic	p. =	1 (1.1)	0	94	NA
13	c.4739 + 24G > T	Intronic	p. =	20 (21.3)	2 (2.1)	94	rs12933120
14	c.5072A > G	Missense	p.Asn1691Ser	1 (1.1)	0	94	NA
15	c.5389C > T	Silent	p. =	1 (1.1)	0	93	NA
15	c.5501A > G	Missense	p.Asn1834Ser	2 (2.2)	0	93	rs111738042
15	c.*8A > G	Intronic	p. =	9 (9.7)	0	93	rs3751839
15	c.*102G > A	Intronic	p. =	1 (1.1)	0	93	NA
15	c.*113C > T	Intronic	p. =	8 (8.6)	0	93	rs76661336

*Het, heterozygous; Hom, homozygous

†Build 133; NA, not applicable

**Figure 1:**
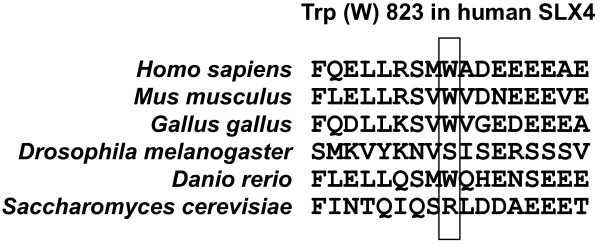
**CLUSTALW-based multi-alignment of human SLX4 and eukaryotic homologs**. The region surrounding human Trp823 is shown.

Having identified rare variants in BrCa familial cases, we next assessed the presence of 10 of these variants in a cohort of controls collected at the same hospital as the cases (see Methods). The selection of these variants was based on the observed low frequency in the 94 BrCa cases and on their identification as missense variations. In addition, a causative prediction was obtained using two algorithms (PolyPhen-2 [26] and SIFT [27]), plus a weighted average of scores (Condel [28]). Seven of these variants were not found in controls and, intriguingly, five of them were predicted to be "deleterious" (Table 2). Among this group, only one amino acid position (Trp823) showed some evolutionary conservation (Figure 1), and the substitution may be disfavored (Trp to Cys) [29]; tumor samples were not available for any case that would have allowed assessment of the existence of loss of heterozygosity at the *SLX4 *locus. Nonetheless, predictions of a deleterious effect should be taken with caution as neutral polymorphisms can frequently be misclassified (from ~15-50% depending on the method [28]). On the other hand, extensive genetic variation in *SLX4 *might reflect an unknown evolutionary pressure or could be related to a similar observation made for other DNA repair-related genes [30].

**Table 2:** Pathological prediction and frequency in controls of selected *SLX4 *missense variants

Exon	Nucleotide change	Amino acid change	dbSNP†	Pathological prediction				Controls tested (*n*)	Number of control carriers (%)
				SIFT (score < 0.05, deleterious)	PolyPhen-2 (false positive rate)	Condel (weighted average of scores)	Condel prediction		
2	c.248G > C	p.Gly83Ala	NA	0.14	0.15	0.15	Neutral	283	0
2	c.421G > T	p.Gly141Trp	NA	0.00	0.86	0.86	Deleterious	284	2 (0.7)
3	c.590T > C	p.Val197Ala	NA	0.48	0.01	0.00	Neutral	284	1 (0.4)
3	c.710G > A	p.Arg237Gln	NA	0.49	0.00	0.38	Neutral	284	4 (1.4)
8	c.1846G > A	p.Val616Met	NA	0.17	0.62	0.80	Deleterious	281	0
12	c.2469G > C	p.Trp823Cys	NA	0.01	1.00	0.97	Deleterious	282	0
12	c.3872C > T	p.Thr1291Met	NA	0.05	0.98	0.99	Deleterious	283	0
12	c.4261A > T	p.Ille1421Phe	NA	0.08	0.77	0.77	Deleterious	285	0
12	c.4409C > T	p.Pro1470Leu	rs72778139	0.02	0.99	0.96	Deleterious	283	0
14	c.5072A > G	p.Asn1691Ser	NA	0.56	0.00	0.01	Neutral	285	0

†Build 133; NA, not applicable

While SLX4 serves as a scaffold for multiple proteins involved in the DNA damage response [16,17], the functional involvement of the Trp823 position, and of the other rare variants not found in controls in this study, is unknown. None of the identified variants changes a critical amino acid residue and there is no data that could suggest an alteration of protein interactions or complexes; however, the Pro1470Leu variant might disrupt a mitotic phosphorylation site at Ser1469 [31,32]. In this context, a *SLX4 *pathological variant linked to BrCa should consist of a hypomorphic mutation that would cause genome instability. Accordingly, SLX4 is a key regulator of the function of structure-specific endonucleases involved in the repair of DNA damage; in particular, proper function of SLX4 is fundamental for repair during replication and for resolving Holliday junctions formed during homologous recombination [16,17,33,34]. Overall, the results of this study do not support the existence of loss-of-function mutations of SLX4 associated with BrCa risk; nonetheless, further genetic analysis in patients and controls, combined with functional assays of specific rare variants, may be warranted.

## Conclusions

The mutational analysis of *SLX4 *in 94 familial BrCa index cases without mutations in *BRCA1 *or *BRCA2 *has revealed extensive genetic variation. Twenty-nine novel single nucleotide variants have been detected, 21 of them showing relatively low allele frequencies: however, none can be linked to a clear alteration of the protein function. Nonetheless, analysis of 10 of these variants failed to detect seven of them in a set of controls. While the results of this study do not support the common existence of *SLX4 *mutations contributing to BrCa risk, additional studies may be warranted.

## Abbreviations

BrCa: Breast cancer; *BRCA1/2: Breast cancer 1/2: early onset *genes; BRCAPRO: *BRCA *carrier prediction model; Condel: Consensus deleteriousness score; dbSNP: Single nucleotide polymorphism database; DNA: Deoxyribonucleic acid; FA: Fanconi anemia; Het: Heterozygous; Hom: Homozygous; PolyPhen: Polymorphism phenotyping; SIFT: Sorting intolerant from tolerant; *SLX4: Structure-specific endonuclease subunit homolog *(*S. cerevisiae*) gene.

## Competing interests

The authors declare that they have no competing interests.

## Authors' contributions

The project was conceived by DS, CL and MAP. The experiments and data analyses were coordinated by JF-R, CL and MAP. The mutational analysis was performed by JF-R and FQ. The clinical and *BRCA1/2 *annotations were performed by IB, AT, LF, JV, MS, AI, ED, GC, JB and CL. The selection of index cases was performed by IB. The manuscript was written by MAP. All authors read and approved the final manuscript.

## Pre-publication history

The pre-publication history for this paper can be accessed here:

http://www.biomedcentral.com/1471-2407/12/84/prepub

## References

[B1] Wang W Emergence of a DNA-damage response network consisting of Fanconi anaemia and BRCA proteins Nat Rev Genet 2007 8 735 748 17768402 10.1038/nrg2159

[B2] D'Andrea AD Susceptibility pathways in Fanconi's anemia and breast cancer N Engl J Med 2010 362 1909 1919 10.1056/NEJMra0809889 20484397 PMC3069698

[B3] Howlett NG Taniguchi T Olson S Cox B Waisfisz Q De Die-Smulders C Persky N Grompe M Joenje H Pals G Biallelic inactivation of *BRCA2 *in Fanconi anemia Science 2002 297 606 609 10.1126/science.1073834 12065746

[B4] Wooster R Bignell G Lancaster J Swift S Seal S Mangion J Collins N Gregory S Gumbs C Micklem G Identification of the breast cancer susceptibility gene *BRCA2* Nature 1995 378 789 792 10.1038/378789a0 8524414

[B5] Seal S Thompson D Renwick A Elliott A Kelly P Barfoot R Chagtai T Jayatilake H Ahmed M Spanova K Truncating mutations in the Fanconi anemia J gene *BRIP1 *are low-penetrance breast cancer susceptibility alleles Nat Genet 2006 38 1239 1241 10.1038/ng1902 17033622

[B6] Levran O Attwooll C Henry RT Milton KL Neveling K Rio P Batish SD Kalb R Velleuer E Barral S The BRCA1-interacting helicase BRIP1 is deficient in Fanconi anemia Nat Genet 2005 37 931 933 10.1038/ng1624 16116424

[B7] Levitus M Waisfisz Q Godthelp BC de Vries Y Hussain S Wiegant WW Elghalbzouri-Maghrani E Steltenpool J Rooimans MA Pals G The DNA helicase BRIP1 is defective in Fanconi anemia complementation group J Nat Genet 2005 37 934 935 10.1038/ng1625 16116423

[B8] Litman R Peng M Jin Z Zhang F Zhang J Powell S Andreassen PR Cantor SB BACH1 is critical for homologous recombination and appears to be the Fanconi anemia gene product FANCJ Cancer Cell 2005 8 255 265 10.1016/j.ccr.2005.08.004 16153896

[B9] Erkko H Xia B Nikkila J Schleutker J Syrjakoski K Mannermaa A Kallioniemi A Pylkas K Karppinen SM Rapakko K A recurrent mutation in *PALB2 *in Finnish cancer families Nature 2007 446 316 319 10.1038/nature05609 17287723

[B10] Reid S Schindler D Hanenberg H Barker K Hanks S Kalb R Neveling K Kelly P Seal S Freund M Biallelic mutations in *PALB2 *cause Fanconi anemia subtype FA-N and predispose to childhood cancer Nat Genet 2007 39 162 164 10.1038/ng1947 17200671

[B11] Rahman N Seal S Thompson D Kelly P Renwick A Elliott A Reid S Spanova K Barfoot R Chagtai T *PALB2*, which encodes a BRCA2-interacting protein, is a breast cancer susceptibility gene Nat Genet 2007 39 165 167 10.1038/ng1959 17200668 PMC2871593

[B12] Meindl A Hellebrand H Wiek C Erven V Wappenschmidt B Niederacher D Freund M Lichtner P Hartmann L Schaal H Germline mutations in breast and ovarian cancer pedigrees establish *RAD51C *as a human cancer susceptibility gene Nat Genet 2010 42 410 414 10.1038/ng.569 20400964

[B13] Vaz F Hanenberg H Schuster B Barker K Wiek C Erven V Neveling K Endt D Kesterton I Autore F Mutation of the *RAD51C *gene in a Fanconi anemia-like disorder Nat Genet 2010 42 406 409 10.1038/ng.570 20400963

[B14] Pelttari LM Heikkinen T Thompson D Kallioniemi A Schleutker J Holli K Blomqvist C Aittomaki K Butzow R Nevanlinna H *RAD51C *is a susceptibility gene for ovarian cancer Hum Mol Genet 2011 20 3278 3288 10.1093/hmg/ddr229 21616938

[B15] Roberts TM Kobor MS Bastin-Shanower SA Ii M Horte SA Gin JW Emili A Rine J Brill SJ Brown GW Slx4 regulates DNA damage checkpoint-dependent phosphorylation of the BRCT domain protein Rtt107/Esc4 Mol Biol Cell 2006 17 539 548 16267268 10.1091/mbc.E05-08-0785 PMC1345688

[B16] Svendsen JM Smogorzewska A Sowa ME O'Connell BC Gygi SP Elledge SJ Harper JW Mammalian BTBD12/SLX4 assembles a Holliday junction resolvase and is required for DNA repair Cell 2009 138 63 77 10.1016/j.cell.2009.06.030 19596235 PMC2720686

[B17] Fekairi S Scaglione S Chahwan C Taylor ER Tissier A Coulon S Dong MQ Ruse C Yates JR Russell P Human SLX4 is a Holliday junction resolvase subunit that binds multiple DNA repair/recombination endonucleases Cell 2009 138 78 89 10.1016/j.cell.2009.06.029 19596236 PMC2861413

[B18] Stoepker C Hain K Schuster B Hilhorst-Hofstee Y Rooimans MA Steltenpool J Oostra AB Eirich K Korthof ET Nieuwint AW SLX4, a coordinator of structure-specific endonucleases, is mutated in a new Fanconi anemia subtype Nat Genet 2011 43 138 141 10.1038/ng.751 21240277

[B19] Kim Y Lach FP Desetty R Hanenberg H Auerbach AD Smogorzewska A Mutations of the *SLX4 *gene in Fanconi anemia Nat Genet 2011 43 142 146 10.1038/ng.750 21240275 PMC3345287

[B20] Landwehr R Bogdanova NV Antonenkova N Meyer A Bremer M Park-Simon TW Hillemanns P Karstens JH Schindler D Dork T Mutation analysis of the *SLX4/FANCP *gene in hereditary breast cancer Breast Cancer Res Treat 2011 130 1021 1028 10.1007/s10549-011-1681-1 21805310

[B21] Euhus DM Smith KC Robinson L Stucky A Olopade OI Cummings S Garber JE Chittenden A Mills GB Rieger P Pretest prediction of *BRCA1 *or *BRCA2 *mutation by risk counselors and the computer model BRCAPRO J Natl Cancer Inst 2002 94 844 851 10.1093/jnci/94.11.844 12048272

[B22] Landi S Moreno V Gioia-Patricola L Guino E Navarro M de Oca J Capella G Canzian F Association of common polymorphisms in inflammatory genes interleukin (IL) 6, *IL8*, tumor necrosis factor alpha, *NFKB1*, and peroxisome proliferator-activated receptor gamma with colorectal cancer Cancer Res 2003 63 3560 3566 12839942

[B23] Sherry ST Ward MH Kholodov M Baker J Phan L Smigielski EM Sirotkin K dbSNP: the NCBI database of genetic variation Nucleic Acids Res 2001 29 308 311 10.1093/nar/29.1.308 11125122 PMC29783

[B24] Consortium GP A map of human genome variation from population-scale sequencing Nature 2010 467 1061 1073 10.1038/nature09534 20981092 PMC3042601

[B25] Reese MG Eeckman FH Kulp D Haussler D Improved splice site detection in Genie J Comput Biol 1997 4 311 323 10.1089/cmb.1997.4.311 9278062

[B26] Adzhubei IA Schmidt S Peshkin L Ramensky VE Gerasimova A Bork P Kondrashov AS Sunyaev SR A method and server for predicting damaging missense mutations Nat Methods 2010 7 248 249 10.1038/nmeth0410-248 20354512 PMC2855889

[B27] Kumar P Henikoff S Ng PC Predicting the effects of coding non-synonymous variants on protein function using the SIFT algorithm Nat Protoc 2009 4 1073 1081 10.1038/nprot.2009.86 19561590

[B28] González-Pérez A Lopez-Bigas N Improving the assessment of the outcome of nonsynonymous SNVs with a consensus deleteriousness score, Condel Am J Hum Genet 2011 88 440 449 10.1016/j.ajhg.2011.03.004 21457909 PMC3071923

[B29] Betts MJ Russell RB Barnes MR, Gray IC Amino acid properties and consequences of subsitutions Bioinformatics for Geneticists 2003 Chichester: John Wiley and Sons 289 316

[B30] Mohrenweiser HW Wilson DM Jones IM Challenges and complexities in estimating both the functional impact and the disease risk associated with the extensive genetic variation in human DNA repair genes Mutat Res 2003 526 93 125 10.1016/S0027-5107(03)00049-6 12714187

[B31] Cantin GT Yi W Lu B Park SK Xu T Lee JD Yates JR Combining protein-based IMAC, peptide-based IMAC, and MudPIT for efficient phosphoproteomic analysis J Proteome Res 2008 7 1346 1351 10.1021/pr0705441 18220336

[B32] Dephoure N Zhou C Villen J Beausoleil SA Bakalarski CE Elledge SJ Gygi SP A quantitative atlas of mitotic phosphorylation Proc Natl Acad Sci USA 2008 105 10762 10767 10.1073/pnas.0805139105 18669648 PMC2504835

[B33] Crossan GP van der Weyden L Rosado IV Langevin F Gaillard PH McIntyre RE Gallagher F Kettunen MI Lewis DY Brindle K Disruption of mouse Slx4, a regulator of structure-specific nucleases, phenocopies Fanconi anemia Nat Genet 2011 43 147 152 10.1038/ng.752 21240276 PMC3624090

[B34] Wechsler T Newman S West SC Aberrant chromosome morphology in human cells defective for Holliday junction resolution Nature 2011 471 642 646 10.1038/nature09790 21399624 PMC3560329

